# Screening for antimicrobial activity of ten medicinal plants used in Colombian folkloric medicine: A possible alternative in the treatment of non-nosocomial infections

**DOI:** 10.1186/1472-6882-6-2

**Published:** 2006-02-17

**Authors:** Jhon J Rojas, Veronica J Ochoa, Saul A Ocampo, John F Muñoz

**Affiliations:** 1 Department of Pharmacy, Universidad de Antioquia, Cll. 67 # 53-108 of. 1-157, Medellín, Colombia

## Abstract

**Background:**

The antimicrobial activity and Minimal Inhibitory Concentration (MIC) of the extracts of *Bidens pilosa *L., *Bixa orellana *L., *Cecropia peltata *L., *Cinchona officinalis *L., *Gliricidia sepium *H.B. & K, *Jacaranda mimosifolia *D.Do*n*, *Justicia secunda *Vahl., *Piper pulchrum *C.DC, *P. paniculata *L. and *Spilanthes americana *Hieron were evaluated against five bacteria (*Staphylococcus aureus, Streptococcus *β *hemolític*, *Bacillus cereus*, *Pseudomonas aeruginosa*, and *Escherichia coli*), and one yeast (*Candida albicans*). These plants are used in Colombian folk medicine to treat infections of microbial origin.

**Methods:**

Plants were collected by farmers and traditional healers. The ethanol, hexane and water extracts were obtained by standard methods. The antimicrobial activity was found by using a modified agar well diffusion method. All microorganisms were obtained from the American Type Culture Collection (ATCC). MIC was determined in the plant extracts that showed some efficacy against the tested microorganisms. Gentamycin sulfate (1.0 μg/ml), clindamycin (0.3 μg/ml) and nystatin (1.0 μg/ml) were used as positive controls.

**Results:**

The water extracts of *Bidens pilosa *L., *Jacaranda mimosifolia *D.Do*n*, and *Piper pulchrum *C.DC showed a higher activity against *Bacillus cereus *and *Escherichia coli *than gentamycin sulfate. Similarly, the ethanol extracts of all species were active against *Staphylococcus aureus *except for *Justicia secunda*. Furthermore, *Bixa orellana *L, *Justicia secunda *Vahl. and *Piper pulchrum *C.DC presented the lowest MICs against *Escherichia coli *(0.8, 0.6 and 0.6 μg/ml, respectively) compared to gentamycin sulfate (0.9 8g/ml). Likewise, *Justicia secunda *and *Piper pulchrum *C.DC showed an analogous MIC against *Candida albicans *(0.5 and 0.6 μg/ml, respectively) compared to nystatin (0.6 μg/ml). *Bixa orellana *L, exhibited a better MIC against *Bacillus cereus *(0.2 μg/ml) than gentamycin sulfate (0.5 μg/ml).

**Conclusion:**

This *in vitro *study corroborated the antimicrobial activity of the selected plants used in folkloric medicine. All these plants were effective against three or more of the pathogenic microorganisms. However, they were ineffective against *Streptococcus *β *hemolytic *and *Pseudomonas aeruginosa*. Their medicinal use in infections associated with these two species is not recommended. This study also showed that *Bixa orellana *L, *Justicia secunda *Vahl. and *Piper pulchrum *C.DC could be potential sources of new antimicrobial agents.

## Background

In developing countries and particularly in Colombia, low income people such as farmers, people of small isolate villages and native communities use folk medicine for the treatment of common infections. These plants are ingested as decoctions, teas and juice preparations to treat respiratory infections[1]. They are also made into a poultice and applied directly on the infected wounds or burns.

When people from these remote communities get an infectious disease, they are usually treated by traditional healers and shamans because of their expertise in such procedures as making diagnoses, treating wounds, setting bones and making herbal medicines. Traditional healers claim that their medicine is cheaper and more effective than modern medicine. Patients of these communities have a reduced risk to get infectious diseases from resistant pathogens than people from urban areas treated with traditional antibiotics. However, if they are treated in a hospital the chance of contracting a nosocomial infection is increased[2].

One way to prevent antibiotic resistance of pathogenic species is by using new compounds that are not based on existing synthetic antimicrobial agents[3]. Traditional healers claim that some medicinal plants such as *bixa spp*. and *bidens spp*. are more efficient to treat infectious diseases than synthetic antibiotics. It is necessary to evaluate, in a scientific base, the potential use of folk medicine for the treatment of infectious diseases produced by common pathogens. Medicinal plants might represent an alternative treatment in non-severe cases of infectious diseases. They can also be a possible source for new potent antibiotics to which pathogen strains are not resistant. [4].

We chose ten species used in folk medicine to determine their antimicrobial activity: *B. pilosa *L. (Asteraceae), *B. orellana *L. (Bixaceae), *C. peltata *L. (Moraceae), *C. officinalis *L. (Rubiaceae), *G. sepium *H.B. & K (Fabaceae), *J. mimosifolia *D.Do*n *(Bignoniaceae), *J. secunda *Vahl. (Acanthaceae), *P. pulchrum *C.DC (Piperaceae), *P. paniculata *L. (Polygalaceae), and *S. americana *Hieron (Asteraceae). In general, these plants are used in folk medicine in the treatment of pharyngitis, gingivitis, bronchitis, infected wounds, topical ulcers, and as antiparasitic agents.

The extract of *B. pilosa *is used in folk medicine as an antihelmintic and protozoacide agent; it also has antiseptic properties[5]. It contains flavonoids [6]. The ethanol extract of the leaves of *B. orellana *possesses antimicrobial activity against Gram (+) microorganisms and *C. albicans *[7]. Also, its leaves have been employed to treat malaria and leishmaniasis[8]. Its seeds contain carotenoids [9]. The ethanol extract of *C. Peltata *has been used as an antibilious, cardiotonic and diuretic agent[10]. In addition, its leaves have been employed against blennorrhea and warts[11,12]. The decoction of the leaves of *C. officinalis *is used to treat amebiasis. Its dry bark is active against *P. falciparum*, and herpes[13]. It contains quinoline alkaloids[14]. Branches and leaves of *G. sepium *are used to reduce fever in children and adults. It has also been used as insecticide and to treat infections produced by *Microsporum canis*, *Trichophyton mentagrophytes*, and *Neisseria gonorrohae *[15]. Its leaves contain triterpene saponins (I and II)[16]. The water extract of *J. mimosifolia *is active against *P. aeruginosa*. Its flowers contain flavones and flavonoids[17]. Its leaves have iridoids, triterpenes, flavones, and steroids[18]. *J. secunda *has been used to disinfect scorpion wounds[19]. *P. pulchrum *is used to disinfect snakebites[20]. Other species exhibit antimicrobial activity against *P. aeruginosa *andC.*albicans *[21]. *Polygala spp*. possesses trypanocidal activity[22,23]. It contains coumarins [24]. Flowers of *S. americana *are used to treat mouth infections and some varieties of herpes. It contains spilantol[25].

Evidently, there are not sufficient scientific studies that confirm the antimicrobial properties of these plants. This study looks into the *in vitro *antimicrobial activity of these plants against six pathogenic microorganisms that cause the most common cases of infectious diseases of poverished communities in Colombia.

## Methods

### Plant material

All the plants were collected by farmers and traditional healers from the Andes and pacific region of Colombia (Antioquia, Chocó and Huila departments). All the species were identified by Professor Francisco Roldan (Instituto de Biología) in the Herbarium of the Universidad de Antioquia (HUA). Voucher numbers and plant organs are shown in Table 1.

### Preparation of plant extracts

The plant extracts were prepared using the modified method of Alade & Irobi [26]. Briefly, three 100 g portions of the dried powdered plant were soaked separately in 500 ml of distilled water, ethanol (98 %) and η-hexane (99 %), for 72 h. Then, each mixture was refluxed followed by agitation at 200 rpm for 1 h. The filtrates obtained were concentrated under vacuum at 40°C to obtain the dry extracts (Table 1). '[see Additional file 1]'.

### Determination of antimicrobial activity

#### Microorganisms used

The test organisms (*S. aureus *ATCC 29737, *S*. β *hemolític *ATCC 10389, *B. cereus *ATCC 14603, *P. aeruginosa *ATCC 25619, *E. coli *ATCC 10536, and *C. albicans *ATCC 53324) were obtained from the microbiology laboratory, College of Medicine of the Universidad de Antioquia.

#### Culture media

The medium used for the activation of the microorganisms was soybean casein broth (SBCB). The following selective agar media were used for the antimicrobial test: Baird-Parker (*S. aureus*), Cetrimide (*P. aeruginosa*), McConkey (*E. coli*), Blood (*S*. β *hemolitic*), Nutritive (*B. cereus*), and Saboraud-dextrose (*C. albicans*). All the culture media were prepared and treated according to the manufacturer guidelines (Becton Dickinson^® ^M.D. USA).

#### Inoculum

The microorganisms were inoculated into SBCB and incubated at 35 ± 2°C for 4 h. The turbidity of the resulting suspensions was diluted with SBCB to obtain a transmittance of 25.0 % at 580 nm. That percentage was found spectrophotometrically comparable to 1 McFarland turbidity standard. This level of turbidity is equivalent to approximately 3.0 × 10^8 ^CFU/ml. The Bausch & Lomb^® ^spectrophotometer, Model Spectronic 20 was used to adjust the transmittance of the working suspensions.

#### Agar diffusion assay

The modified agar well diffusion method of Perez *et al*., [27] was employed. Each selective medium was inoculated with the microorganism suspended in SBCB. Once the agar was solidified, it was punched with a six millimeters diameter wells and filled with 25 μL of the plants extracts and blanks (ethanol, distilled water, and hexane). The concentration of the extracts employed was 25 μg/ml. Simultaneously, gentamycin sulfate (*S. aureus*, *P. aeruginosa*, *E. coli*, and *B. cereus*), clindamycin (*S*. β *hemolytic*), and nystatin (*C. albicans*) were used as positive controls at a concentration of 1.0, 0.3 and 1.0 μg/ml respectively. The dilution medium for the positive controls was sterile distilled water. The test was carried out by triplicate. The plaques were incubated at 35 ± 2°C for 24 h, except for *C. albicans *which was incubated at 29 ± 2°C. The antimicrobial activity was calculated by applying the expression:

%RIZD=(IZD sample−IZD negative control)IZD antibiotic standard×100%     (1)
 MathType@MTEF@5@5@+=feaafiart1ev1aaatCvAUfKttLearuWrP9MDH5MBPbIqV92AaeXatLxBI9gBaebbnrfifHhDYfgasaacH8akY=wiFfYdH8Gipec8Eeeu0xXdbba9frFj0=OqFfea0dXdd9vqai=hGuQ8kuc9pgc9s8qqaq=dirpe0xb9q8qiLsFr0=vr0=vr0dc8meaabaqaciaacaGaaeqabaqabeGadaaakeaacqGGLaqjcqqGsbGucqqGjbqscqqGAbGwcqqGebarcqGH9aqpdaWcaaqaaiabcIcaOiabbMeajjabbQfaAjabbseaejabbccaGiabbohaZjabbggaHjabb2gaTjabbchaWjabbYgaSjabbwgaLjabgkHiTiabbMeajjabbQfaAjabbseaejabbccaGiabb6gaUjabbwgaLjabbEgaNjabbggaHjabbsha0jabbMgaPjabbAha2jabbwgaLjabbccaGiabbogaJjabb+gaVjabb6gaUjabbsha0jabbkhaYjabb+gaVjabbYgaSjabcMcaPaqaaiabbMeajjabbQfaAjabbseaejabbccaGiabbggaHjabb6gaUjabbsha0jabbMgaPjabbkgaIjabbMgaPjabb+gaVjabbsha0jabbMgaPjabbogaJjabbccaGiabbohaZjabbsha0jabbggaHjabb6gaUjabbsgaKjabbggaHjabbkhaYjabbsgaKbaacqGHxdaTcqaIXaqmcqaIWaamcqaIWaamcqGGLaqjcaWLjaGaaCzcamaabmaabaGaeGymaedacaGLOaGaayzkaaaaaa@826A@

Where RIZD is the percentage of relative inhibition zone diameter and IZD is the inhibition zone diameter (mm). Equation (1) compensates the possible effect of the solvent (blank) other than water on the IZD. The resulting IZD of the samples were either higher than or equal to the IZD of the blanks. Therefore, the obtained percentages were positive (Table 1). '[see Additional file table1]'. The test was considered negative (-) when the IZD of the sample was equal to the IZD of the blank.

#### Minimal inhibitory concentration (MIC) evaluation

The MIC was evaluated on plant extracts that showed antimicrobial activity. This test was performed at four concentrations of each extract (6.3, 12.5, 25, 50 μg/ml) employing the same modified agar well diffusion method. Calculations of MIC were determined by regression analysis using the software STATGRAPHICS^® ^v. 4 (Table 2). '[see Additional file table1]'.

#### Phytochemical screening

The method of Martinez & Valencia [28] was implemented to identify the general phytochemical groups of compounds in the extracts (Table 1). '[see Additional file table1]'. The test for amino acids was conducted by dissolving 10 mg of dry extracts in 1 ml of ethanol and adding 1 droplet of ninhidrine reagent. For flavonoids, Shimoda's test was adopted (15 mg of dry extract was dissolved in 1 ml of ethanol, concentrated HCl, and magnesium turnins were added). Anthocyanins were identified by adding 1 ml of boiling water, 0.5 ml of 37 % HCl to 10 mg of dry extract. The solution was heated at 100°C, cooled and added 0.4 ml of amylic alcohol.

The test for phenolic compounds was carried out by dissolving 10 mg of dry extract in 1 ml of 1 % ferric chloride solution. For tannins, 1 ml of the gelatin reagent was added to 1 ml of the filtered aqueous extract. Quinones were identified by extracting 10 ml of the aqueous extract with dichloromethane, evaporating the organic phase, and adding 5 ml of ethanol, 1 ml of hydrogen peroxide 5 % and 1 ml of sulfuric acid 50 % respectively. The solution was heated, cooled, extracted with benzene and 1 ml of ammonia solution added.

Cardiac glycosides were identified by evaporating 1 ml of the organic phase, dissolving the residue in 1 ml of ethanol and adding 0.5 ml of Kedde's reagent. For triterpenoids and steroids, 0.5 ml of acetic anhydride and 1 droplet of 37 % sulfuric acid solution were added to 0.5 ml of the organic phase. The test for alkaloids was carried out by adding 0.5 ml of the aqueous extract into four test tubes; boiled, filtered and one droplet of the reagents of Mayer, Valser, Dragendorff and ammonium Reineckate was added respectively.

### Statistical analysis

All values are expressed as means ± standard deviation (Table 2). '[see Additional file table1]'. The MIC data for each microorganism were analyzed using one-way analysis of variance (ANOVA) and the differences among group means were analyzed using the Dunnett's multiple comparisons test. P value < 0.05 was considered as significant. The software MINITAB^® ^was employed for the statistical analysis.

## Results and discussion

All the plants showed antimicrobial activity in regards to at least three microorganisms tested (Table 1). '[see Additional file table1]'. The ethanol extracts of *B. orellana *(seeds), *G. sepium*, *J. mimosifolia *and *P. pulchrum *were the most active against the microorganisms studied. In some cases, the three extracts of the same plant had antimicrobial activity against the same microorganism. For instance, the three extracts of *J. mimosifolia *were active against *B. cereus*. This possibly means that the compound responsible for the antimicrobial activity was present in each extract at a different concentration.

All the plants exhibited different kinds of secondary metabolites. In particular, *J. mimosifolia *presented the highest yield of extractable substances in the water extract (15.0%). This result indicates that decoction is a good method to extract anthocyanins, phenolic compounds, and alkaloids found in this species. These compounds could be responsible for its antimicrobial activity against *B. cereus *and *E. coli*. Former studies of the water extract revealed antimicrobial activity against *P. aeruginosa *[17]. However, we did not find antimicrobial activity against this pathogenic microorganism. Furthermore, the water extract of *J. mimosifolia*, and *P. pulchrum *showed a higher activity (compared to the gentamycin sulfate) against *B. cereus*. The water extract of *P. pulchrum *also showed antimicrobial activity against *S. aureus*.

Ethanol extracts exhibited a higher degree of antimicrobial activity as compared with water and hexane extracts fractions. This finding is correlated with the medicinal preparations that use rum and liquor to extract the active plant components.

*E. coli, B. cereus *and *S. aureus *were the most susceptible bacteria to all plant extracts. On the contrary, *S*. β *hemolytic*, *P. aureginosa *and *C. albicans *were the most resistant microorganisms. None of the extracts was more active against *S*. β *hemolytic *than the positive control (clindamycin). Likewise, the ethanol extract of *J. secunda *was the only extract active against *P. aeruginosa*. Only three plants (*J. secunda, P. pulchrum *and *P. paniculata*) were the most active against *C. albicans*. This result is in accord with formers studies executed on *Piper spp*. [21].

Steroids and anthocyanins of *B. orellana *(seeds) could be responsible for their antimicrobial activity against *S. aureus, B. cereus *and *E. coli*. Similarly, the presence of steroids and amino acids in *C. peltata *could correspond to its high antimicrobial activity exhibited against *E. coli. B. pilosa *showed a low activity against *S. aureus *and *B. cereus*. However, some studies established that the methanol extract of this plant is highly active against *S. aureus*, *S. epidermidis *and *B. subtilis *[29].

*C. officinalis *was the species that exhibited the greatest variety of secondary metabolites. It also showed antimicrobial activity against all the pathogens studied. Similarly, *S. americana *presented antimicrobial activity against all the microorganisms studied except for *C. albicans*. Alkaloids and steroids found in this plant might account for this. Former studies associated the alkaloid spilantol with its biological activity [21]. This plant also showed the lowest yield of extractable solids among all the plants (0.02%).

Table 2 shows that *J. secunda *and *P. pulchrum *presented similar MICs against *C. albicans *(0.5 and 0.6 μg/ml, respectively) compared to nystatin (0.6 μg/ml). Moreover, these two plants manifested a better MIC against *E. coli *(0.6 μg/ml) than gentamycin sulfate (0.9 μg/ml). However, there was no statistical difference between MICs of seven plants and gentamycin sulfate. *J. secunda and B. orellana *were the only plants that exhibited a similar MIC against *P. aeruginosa *(1.3 and 4.8 μg/ml) compared to gentamycin sulfate (0.3 μg/ml).

*B. orellana *L, *J. secunda *and *P. pulchrum *presented the lowest MICs against *E. coli *(0.8, 0.6 and 0.6 μg/ml, respectively) compared to gentamycin sulfate (0.9 μg/ml). Likewise, *B. orellana *manifested the lowest value of MIC against *B. cereus *(0.2 μg/ml) compared to gentamycin sulfate (0.5 μg/ml). However, for *B. cereus *there was no statistical difference between MICs of eight plants and gentamycin.

## Conclusion

All the extracts showed varying degrees of antimicrobial activity on the microorganisms tested. Some of these plants were more effective than traditional antibiotics to combat the pathogenic microorganisms studied. The chance to find antimicrobial activity was more apparent in ethanol than water extracts of the same plants. Three species (*B. orellana, J. secunda *and *P. pulchrum *presented the lowest MIC compared to the antibiotic standard. These plants could be a source of new antibiotic compounds. Further work is needed to isolate the secondary metabolites from the extracts studied in order to test specific antimicrobial activity.

This *in vitro *study demonstrated that folk medicine can be as effective as modern medicine to combat pathogenic microorganisms. The millenarian use of these plants in folk medicine suggests that they represent an economic and safe alternative to treat infectious diseases. However, none of the plants are recommended in the treatment of infections produced by *S*. β *hemolitic *and *P. aeruginosa*.

## List of abbreviations

ATCC – American Type Culture Collection

*B. cereus *– *Bacillus cereus*

*B. pilosa *– *Bidens pilosa *L

*B. subtilis *– *Bacillus subtilis*

*B. orellana *– *Bixa orellana *L.

C. albicans-Candida albicans

*C. peltata *– *Cecropia peltata *L.

*C. officinalis *– *Cinchona officinalis *L.

*E. coli *– *Escherichia coli*

*G. sepium *– *Gliricidia sepium *H.B. & K

*J. secunda *– *Justicia secunda *Vahl

*J. mimosifolia *– *Jacaranda mimosifolia *D.Do*n*

*P. aeruginosa *– *Pseudomonas aeruginosa*

*P. pulchrum *– *Piper pulchrum*

*P. paniculata *– *Polygala paniculata *L.

SBCB-Soybean casein broth

*S*. β *hemolitic *– *Streptococcus *β *hemolític*

*S. aureus *– *Staphylococcus aureus*

*S. epidermidis *– *Staphylococcus epidermidis*

*S. americana *– Spilanthes americana Hieron

## Competing interests

The author(s) declare that they have no competing interests.

## Authors' contributions

JJR conceived and designed the study, performed the MIC tests, analyzed the data obtained and drafted this paper. JFM and SAO interviewed local healers and farmers; they also performed the phytochemical screening and the antimicrobial tests. VJO Participated in the extraction, susceptibility testing and analysis of data. All authors read and approved the final manuscript.

## Pre-publication history

The pre-publication history for this paper can be accessed here:



## Supplementary Material

Additional file 1 Table 1 – Antimicrobial activity and phytochemicals screening of the plants studied. Table 2 – Minimum Inhibitory Concentration of the plants studied. Click here for file
