# Supplementary material for: Screening for antimicrobial activity of ten medicinal plants used in Colombian folkloric medicine: A possible alternative in the treatment of non-nosocomial infections
Source: BMC Complement Altern Med. 2006 Feb 17;6:2. doi: 10.1186/1472-6882-6-2 (PMC1395329; doi:10.1186/1472-6882-6-2)
Supplement: Additional file 1 — Table 1 – Antimicrobial activity and phytochemicals screening of the plants studied. Table 2 – Minimum Inhibitory Concentration of the plants studied. [file 1472-6882-6-2-S1.rtf]

Tables

Table 1- Antimicrobial activity and phytochemicals screening of the plants studied
Botanic name, (voucher code)	Family	Local name	Organ
 tested1	Phytochemical screening2	Fraction extract3	Yield
(%w/w)	% RIZD ( mean +/-SD)4	
							Staphylococcus aureus	Bacillus Cereus	Streptococcus b hemolytic	Escherichia coli	Pseudomonas aeruginosa	Candida albicans	
Bidens pilosa L.
(RF2568)	Asteraceae	Chipaca,
Masequia	LE,SB,FW	AA,PC,TE, AC,FL,AL.	I 	9.0	(-)	(-)	(-)	104.9±0.3	(-)	(-)	
					II 	0.6	66.7±0.5	78.8±5.0	(-)	132.9±0.8	(-)	(-)	
					III 	0.3	(-)	54.5±0.3	(-)	97.9±0.3	(-)	158.0±0.6	
Bixa orellana L.
(RF1203)	Bixaceae	Achiote	LE	PC, ST, FL, AC, TA.	I 	6.50	(-)	(-)	(-)	83.9±0.7	(-)	(-)	
					II 	9.50	133.3±0.5	103.3±0.9	(-)	104.6±3.0	52.0±0.0	(-)	
					III	0.81	(-)	(-)	(-)	(-)	(-)	(-)	
Bixa orellana L.
(RF1204)	Bixaceae	Achiote	SE	ST, AC.	I	9.40	(-)	(-)	(-)	(-)	(-)	(-)	
					II 	6.04	138.0±0.6	107.3±0.5	33.3±0.3	128±0.3	(-)	(-)	
					III 	1.21	103.3±0.0	102.1±0.3	(-)	118.9±0.0	55.7±0.6	(-)	
Cecropia peltata L.
(RF1205)	Moraceae	Yarumo	LE	AA, ST.	I 	10.4	(-)	(-)	(-)	76.9±0.0	(-)	(-)	
					II 	2.98	78.0±0.6	83.0±0.3	(-)	104.9±0.0	(-)	(-)	
					III 	3.95	(-)	(-)	24.3±0.3	103.3±0.5	(-)	94.7±0.0	
Cinchona officinalis L.
(RF1203)	Rubiaceae	Quina	SB	AC, PC, TA, ST, CG, QU, AL.	I	8.90	(-)	(-)	(-)	(-)	(-)	(-)	
					II 	6.01	138±1.0	101.8±0.6	25.8±0.6	(-)	47.8±0.0	84.2±0.0	
					III	1.05	(-)	112.3±0.3	(-)	107.4±0.3	(-)	(-)	


Table 1- (Continuation)
Botanic name, (voucher code)	Family	Local name	Organ
 tested1	Phytochemical screening2	Fraction extract3	Yield
(%w/w)	% RIZD (mean +/-SD)4	
							Staphylococcus aureus	Bacillus Cereus	Streptococcus b hemolytic	Escherichia coli	Pseudomonas aeruginosa	Candida albicans	
Gliricidia sepium H.B. & K
(RF1206)	Fabaceae	mataraton	LE	PC, ST.	I	14.0	(-)	(-)	(-)	(-)	(-)	(-)	
					II 	9.40	133.3±0.0	104.8±0.3	32.5±0.0	114.0±0.3	(-)	(-)	
					III 	1.54	(-)	121.2±0.0	(-)	103.5±0.0	(-)	(-)	
 Jacaranda mimosifolia D.Don
(RF1207)	Bignoniaceae	Gualanday	LE	PC, AC, AL.	I 	15.0	(-)	100.4±0.6	(-)	87.4±0.6	(-)	(-)	
					II 	10.0	146.7±0.0	138.8±0.5	(-)	(-)	(-)	(-)	
					III 	0.92	(-)	106.2±0.3	(-)	107.8±0.5	(-)	(-)	
Justicia secunda Vahl.
(RF6408)	Acanthaceae	singamochila	LE,SB	AA,FL,TE, AC, GC	I	2.1	(-)	(-)	(-)	100.5±0.6	(-)	(-)	
					II 	0.7	(-)	(-)	(-)	103.9±0.3	123.9±0.3	178.9±0.0	
					III 	1.3	(-)	60.6±0.0	(-)	(-)	(-)	(-)	
Piper pulchrum C.DC
(RF6430)	Piperaceae	desvanecedora	LE, SB	AA, PC, TE, AC,TA,FL.	I 	7.5	96.7±0.3	133.3±0.0	(-)	86.0±0.5	(-)	(-)	
					II 	3.4	90.3±0.5	103.0±0.0	30.0±0.4	108.4±0.6	(-)	(-)	
					III 	1.1	(-)	(-)	28.8±0.3	163.6±1.0	(-)	168.4±0.0	
Polygala paniculata L.
(RF3467)	Polygalaceae	Sarpoleta,	WP	AA,PC,TE,AC,TA,AL.	I 	4.8	(-)	(-)	(-)	83.9±0.5	44.8±0.6	(-)	
					II 	7.0	90.0±0.3	(-)	30±0.3	122.4±0.0	(-)	179.0±0.0	
					III 	0.1	(-)	(-)	(-)	129.4±0.0	(-)	(-)	


Table 1- (Continuation)


Botanic name, (voucher code)	Family	Local name	Organ tested1	Phytochemical screening2	Fraction extract3	Yield
(%w/w)	% RIZD (mean +/-SD)4	
							Staphylococcus aureus	Bacillus Cereus
	Streptococcus b hemolytic
	Escherichia coli
	Pseudomonas 
aeruginosa
	Candida albicans
	
Spilanthes americana Hieron
(RF1208)	Asteraceae	Yuyo quemado	WP	ST, AL.	I 	0.14	(-)	(-)	25.0±0.4	(-)	(-)	(-)	
					II 	0.10	100.7±0.6	60.6	30.0±0.8	83.9±0.0	(-)	(-)	
					III 	0.02	(-)	(-)	45.7±0.9	105.6±0.0	52.2±0.3	(-)	
Positive controls	Gentamycin
(1.0 µg/ml)	NA	NA	NA	NA	NA	15.0±0.3	16.5±0.0	NA	9.3±0.3	23.0±0.0	NA	
	Clindamycin sulfate (0.3 µg/ml)	NA	NA	NA	NA	NA	NA	NA	40.0±0.0	NA	NA	NA	
	Nystatin (1.0 µg/ml)	NA	NA	NA	NA	NA	NA	NA	NA	NA	NA	9.5±0.3	
Negative controls	I,II,III	NA	NA	NA	NA	NA	(-)	(-)	(-)	(-)	(-)	(-)	

1LE= leaves, SB= stem bark, SE= seeds, WP= whole plant.
2AL= Alkaloids, AA= Amino acids, AC= Anthocyanins CG= Cardiac glycosides, FL=Flavonoids, PC= Phenolic compounds, QU= Quinones, ST= Steroids, TA= Tannins, TE= Terpenoids.
3I = distilled water, II = ethanol, III = hexane.
4RIZD =Percentage of relative inhibition zone diameter at 25 µg/ml (compared to the respective antibiotic standard). Values are means of three replications.
NA = not applicable. (-) = no inhibition of growth at the concentrations tested.


Table 2- Minimum Inhibitory Concentration of the plants studied

Plant species
(organ tested) 1	Fraction extract2	Minimum inhibitory concentration (ìg /ml) 3	
		Staphylococcus aureus	Bacillus Cereus	Streptococcus b hemolytic	Escherichia coli	Pseudomonas aeruginosa	Candida albicans	
Bidens pilosa L.	I	NT	NT	NT	11.1±0.8	NT	NT	
	II	36.3±6.3	27.7±5.8	NT	8.2±0.5	NT	NT	
	III	NT	NT	NT	47.1±4.3	NT	*1.8±0.0	
Bixa orellana L.(LE)	I	NT	NT	NT	15.3±2.1	NT	NT	
	II	*2.1±0.1	*0.2 ±0.1	NT	*2.1±0.7	*4.8±1.2	NT	
Bixa orellana L. (SE)	II	*1.8±0.2	*0.7±0.0	10.6±0.3	*2.0±0.1	NT	NT	
	III	*1.6±0.3	*1.6±0.0	NT	*0.8±0.1	5.7±2.1	NT	
Cecropia peltata L. (LE)	I	NT	NT	NT	54.7±6.8	NT	NT	
	II	16.6±0.8	14.9±3.4	NT	*3.6±0.2	NT	NT	
	III	NT	NT	20.2±2.9	*2.9±0.6	NT	21.5±6.3	
Cinchona officinalis L. (SB)	II	8.2±0.9	*4.5±0.3	11.7±0.0	NT	16.4±0.3	13.5±3.5	
	III	NT	10.3±1.4	NT	*2.0±0.3	NT	NT	
Gliricidia sepium H.B. & K  (LE)	II	7.8±0.5	6.8±0.9	12.8±0.0	*2.0±0.0	NT	NT	
	III	NT	10.8±1.3	NT	*1.4±0.1	NT	NT	
Jacaranda mimosifolia D.Don (LE)	I	NT	*4.0±0.6	NT	14.4±3.2	NT	NT	
	II	*2.8±0.7	*3.3±0.0	NT	NT	NT	NT	
	III	NT	*2.9±0.3	NT	2.3±0.1	NT	NT	
Justicia secunda Vahl. (LE, SB)	I	NT	NT	NT	*1.8±0.1	NT	NT	
	II	NT	NT	NT	*0.6±0.0	*1.3±0.1	*0.5±0.0	
	III	NT	78.6±9.3	NT	NT	NT	NT	
Piper pulchrum C.DC (LE, SB)	I	32.1±4.1	9.8±0.2	NT	NT	NT	NT	
	II	15.7±2.1	*3.4±1.4	17±3.2	*0.6±0.0	NT	NT	
	III	NT	NT	*5.7±1.2	*1.0±0.1	NT	*0.6±0.1	
Polygala paniculata L. (WP)	I	NT	NT	NT	24.5±2.5	28.3±5.2	NT	
	II	NT	NT	35±6.2	9.8±0.2	NT	11.6±0.4	
	III	NT	NT	NT	14.9±0.0	NT	NT	
Spilanthes americana Hieron (LE)	I	NT	NT	18.6±3.3	NT	NT	NT	
	II	*4.6±0.6	18.6±3.2	15.1±2.1	15.3±3.4	NT	NT	
	III	NT	NT	*2.8±0.2	*4.3±0.5	11.9±1.3	NT	
Positive 
controls	Gentamycin sulfate	NA	0.4±0.0	0.5±0.0	NT	0.9±0.0	0.3±0.0	NT	
	Clindamycin	NA	NT	NT	0.1±0.0	NT	NT	NT	
	Nystatin	NA	NT	NT	NT	NT	NT	0.6±0.0	

1LE= leaves, SB= stem bark, SE= seeds, WP= whole plant, WE= water extract. 
2I = distilled water, II = ethanol, III = hexane.
3Values are means of three replications +/- standard deviation. NT = not tested; no inhibition was showed in the preliminary assay (Table 1).*No statistical difference between this MIC and the antibiotic standard (Dunnett's comparisons test).
